# Surface modification of poly(vinylidene fluoride) hollow fibre membranes for biogas purification in a gas–liquid membrane contactor system

**DOI:** 10.1098/rsos.171321

**Published:** 2017-11-15

**Authors:** Pengrui Jin, Chuan Huang, Jiaxiang Li, Yadong Shen, Liao Wang

**Affiliations:** 1 State Key Laboratory of Coal Mine Disaster Dynamics and Control, Chongqing University, Chongqing 400044, People's Republic of China; 2 College of Resources and Environmental Science, Chongqing University, Chongqing 400044, People's Republic of China

**Keywords:** PVDF hollow fibre membrane, spray deposition, superhydrophobic

## Abstract

The wetting of hollow fibre membranes decreases the performance of the liquid–gas membrane contactor for CO_2_ capture in biogas upgrading. To solve this problem, in this work, a poly(vinylidene fluoride) (PVDF) hollow fibre membrane for a liquid–gas membrane contactor was coated with a superhydrophobic layer composed of a combination of hydrophobic SiO_2_ nanoparticles and polydimethylsiloxane (PDMS) by the method of spray deposition. A rough layer of SiO_2_ deposited on the PVDF membrane resulted in an enhanced surface hydrophobicity. The surface structure of the pristine PVDF significantly affected the homogeneity of the generated SiO_2_ layer. A uniform surface coating on the PVDF upper layer resulted from the presence of micrometre and nanometre-sized roughness on the surface of the PVDF membrane, which was achieved with a SiO_2_ concentration of 4.44 mg ml^−1^ (0.2 g/45 ml) in the coating solution. As a result, the water contact angle of the modified surface was recorded as 155 ± 3°, which is higher than that of the pristine surface. The high contact angle is advantageous for reducing the wetting of the membrane. Additional mass transfer resistance was introduced by the superhydrophobic layer. In addition, continuous CO_2_ absorption tests were carried out in original and modified PVDF hollow fibre membrane contactors, using monoethanolamine (MEA) solution as the absorbent. A long-term stability test revealed that the modified PVDF hollow fibre membrane contactor was able to outperform the original membrane contactor and demonstrated outstanding long-term stability, suggesting that spray deposition is a promising approach to obtain superhydrophobic PVDF membranes for liquid–gas membrane absorption.

## 1. Introduction

With the rapid development of industry, low-carbon renewable energy has attracted considerable interest due to its potential for reduced emissions of greenhouse gases. Biogas is one source of alternative energy that is generated from anaerobic fermentation of organic materials (e.g. landfill, waste, compost and sewage). Typically, raw biogas consists of methane (CH_4_, 40%–75%), carbon dioxide (CO_2_, 15%–60%) and traces of hydrogen sulfide [1]. In addition to being an alternative fuel for large-scale operations, biogas can also be used as fuel for vehicles, for which demand has risen significantly over the past few decades [2]. In these applications, purification steps are essential to meet specifications, because the exothermic value of biogas is reduced by the large amount of CO_2_ present. Moreover, the presence of acidic CO_2_ may corrode equipment. Therefore, it is of considerable importance to seek an efficient, stable and economically viable approach to remove CO_2_ from biogas. Several technologies are available for the removal of CO_2_ from the biogas, such as absorption (physical or chemical solvent), solid adsorption, cryogenics and membrane separation [3–5]. The conventional pressurized liquid scrubbing process has been considered to be a simple and cheap technology and is applied in more than 53% of biogas purification processes among industrial facilities [6]. However, the conventional absorption processes have unavoidable disadvantages such as flooding, foaming, entraining, channelling and high capital costs; these problems are caused by the direct contact between gas and liquid. Therefore, researchers need to develop better processes to remove CO_2_ from biogas.

Gas–liquid membrane contactors (GLMCs) are a new and effective strategy for gas absorption that integrates membrane separation and liquid absorption. Compared with traditional absorption processes, GLMC combines the advantages of both liquid absorption (i.e. low cost and high selectivity) and membrane separation (i.e. high surface-area-to-volume ratio, modularity, compact equipment) methods [7]. Some researchers have reported that the specific surface area of GLMC is 1 to 25 times more than that of conventional contactors; therefore, GLMC can provide a flexible modular device with small size [8]. Furthermore, the independent control of gas and liquid flow rates can help in avoiding the aforementioned operational problems associated with conventional absorption processes [9].

Although GLMC provides advantages that conventional absorption techniques do not possess, additional mass transfer resistance is introduced due to the presence of the membrane. The hydrophobic membrane pores used in GLMC should be filled with gas, which is equivalent to the non-wetting mode. However, after short periods of operation, the absorbents can penetrate into the pores of the hydrophobic membrane, resulting in a rapid decrease in CO_2_ absorption flux. Zhang *et al*. [10] studied CO_2_ capture with deionized water and 2 mol l^−1^ monoethanolamine (MEA) solutions as absorbents in a poly(vinylidene fluoride) (PVDF) hollow fibre membrane contactor experimentally. It was found that CO_2_ flux decreased approximately 54.5% when MEA was used as the absorbent due to membrane wetting compared with the non-wetted mode. The simulation results of Wang *et al*. [11] also showed that even if only 5% of the membrane pores were wetted, the overall mass transfer coefficient may be reduced by 20%. Therefore, in order to ensure the efficiency of the absorption process and the stability of long-term operation, further experimental investigations should be carried out to mitigate the problem of membrane wetting.

The problem of membrane pore wetting can be resolved by increasing the hydrophobicity of membrane. Most of the studies on anti-wetting have been focused on CO_2_ absorption using hydrophobic membranes, but it should be noted that hydrophobic membranes are also easily wetted due to the absorbent molecules diffusing into the membrane polymers during CO_2_ absorption processes [12,13]. Inspired by the performance of superhydrophobic surfaces in resisting wetting, the fabrication of superhydrophobic membranes rather than hydrophobic membranes is presumed to be a potential solution for the problem of membrane wetting [14]. Yu *et al*. [15] fabricated a superhydrophobic ceramic membrane for gas–liquid membrane contactor application that demonstrated superior anti-wetting and anti-fouling features to the polypropylene (PP) membrane due to its superhydrophobic surface.

Superhydrophobicity arises not only from low-energy molecules but also from well-defined surface roughness. Surfaces that have contact angles greater than 150° can be considered to be superhydrophobic. It has been established that the contact angle of a flat, non-gap surface of contiguous hexagonal-packed -CF_3_ groups is 120° [16]. Therefore, superhydrophobic surfaces can be attained only by increasing the porosity [17] and roughness [18].

Recently, materials with superhydrophobic surfaces have become essential components due to their wide range of applications. In general, high water contact angle surfaces can be fabricated by two approaches: (i) the introduction of micro- and nano-scale roughness on hydrophobic surfaces to increase the air gap and (ii) the addition of low surface energy materials to rough surfaces. Various techniques for fabricating superhydrophobic surfaces based on these two approaches have been reported, such as the fluorination of polymer surfaces using plasma [19–21], chemical vapour deposition [22–24], phase separation [25–27], the sol–gel process [28–30], layer-by-layer deposition [31–33] and template synthesis [34]. These methods employ expensive equipment and materials, and the complicated requisite approaches limit their applications. Among them, the multilayer deposition of nanoparticles is one of the most common approaches used to impart superhydrophobicity to surfaces. Razmjou *et al*. [35] successfully obtained superhydrophobic PVDF membranes with a contact angle of 163° ± 3° through low-temperature hydrothermal treatment of TiO_2_ coatings inducing multi-level roughness. Privett *et al*. [36] used silica colloids and fluorinated silane dry gels to synthesize silica-colloid-based superhydrophobic surfaces that reduced bacterial adherence. However, these reported methods require complex processes and involve multiple steps, which limits their commercial application.

Spray deposition, which has been widely used in spraying and drawing industries, is a simple and common method. Hwang *et al*. [37] applied such a spray deposition method to produce a transparent and remarkable superhydrophobic surface. Ogihara *et al*. [38] obtained a superhydrophobic paper by spraying an ethanol suspension containing silica nanoparticles at room temperature and atmospheric pressure. Zhang *et al*. [39] fabricated a superhydrophobic flat membrane by controlling the spray conditions and suspension concentration. It is expected that future research on superhydrophobic membranes will extensively use the spray deposition technology, because this technique can be applied to large-scale applications and various substrates. However, it is difficult to modify a hollow fibre membrane having a diameter of 1.1 mm when compared with the aforementioned successfully modified substrates including glass, paper and flat sheet membranes. Moreover, no attention has been given to the coating of PVDF hollow fibre membranes, which function as membrane gas contactors for biogas upgrading, using the spray deposition technique.

In this work, a rough layer of silica was adhered to a porous PVDF fibre membrane surface using a simple spray deposition method. SiO_2_ nanoparticles and polydimethylsiloxane (PDMS) were dispersed in methyl ethyl ketone (MEK). After the spray treatment, the substrate (PVDF) surface was evenly covered with a coarse silica coating. Various methods were used to study the properties of the modified membrane. The morphology of the surface-modified membranes was characterized by scanning electron microscopy (SEM) and atomic force microscopy (AFM). In addition, the wettability of the membranes was obtained by contact angle measurements. The performance of the membrane in GLMC during CO_2_ absorption with deionized water and MEA solution was investigated. The objective of this work is to study the feasibility of improving the durability and performance of PVDF hollow fibre membrane contactors by spray deposition for CO_2_ capture in biogas upgrading.

## 2. Experimental

### 2.1. Materials

Commercial-grade PVDF hollow fibres of 0.8 mm inside diameter (lumen), 0.15 mm thickness and 0.2 µm pore size were purchased from Tianjin Haizhihuang Technology Co., Ltd, China. Methyl ethyl ketone (MEK) and MEA were procured from Chongqing Chuandong Chemical Industry Co., Ltd, China. Two-component polydimethylsiloxane (PDMS, Sylgard 184, average size 7 nm) was procured from Shanghai Deji Trade Co., Ltd, China. AEROSIL R106, which is a hydrophobic fumed silica with a surface area of 300 m^2^ g^−1^ based on hydrophilic fumed silica and treated with octamethylcyclotetrasiloxane (D4), was obtained from Guangzhou Heqian Trade Co., Ltd, China. All the experimental chemicals were analytically pure reagents.

PDMS (Sylgard 184) which can be used as potting glue is a two-component silicon elastomer. When Sylgard 184 A and Sylgard 184 B are thoroughly mixed, the mixture cures to a flexible elastomer, which can adhere SiO_2_ nanoparticles to the PVDF hollow fibre membrane.

### 2.2. Surface modification of membranes

Approximately 0.08 to 0.6 g of silica (R106) was placed in a conical flask containing 35 ml of MEK. After stirring (10 min) and sonicating (30 min), the PDMS precursor (Sylgard 184 A) was dissolved into the solution, which was then irradiated by ultrasound for 1 h to form solution I. Solution II was formed by mixing the PDMS hardener (Sylgard 184 B) with 10 ml of MEK. Finally, solution I and solution II were mixed to yield a spray solution at room temperature. The obtained suspensions were sprayed onto the desired substrates at 1 to 2 bar at 0.2 ml s^−1^ from a 10 cm working distance using an artist's airbrush (nozzle diameter = 0.2 mm). The modified PVDF hollow fibre membrane was dried at room temperature for 24 h. In order to determine the optimum modification conditions, a series of samples were prepared under different modification conditions. The solution formulation used in the modification is presented in table 1.

**Table 1.** Solution formulation used in membrane modification.

modification solution formulation	value
spraying solution composition (g R106/45 ml solution)	R106: PDMS = 10:11 R106 = 0.08, 0.2, 0.4, 0.6

### 2.3. Membrane characterization

The surface morphology of the membranes before and after the modification, as well as the membrane cross sections, was examined by scanning electron microscopy (FEG-SEM, Nova 400, FEI, USA) operated at 2 kV. In order to prevent charging of the PVDF hollow fibre membrane, it was placed in a vacuum chamber and gold coated for 20 s. The distribution of silicon in the PVDF hollow fibre membrane was studied by energy-dispersive spectroscopy (EDS) and an X-ray detector mounted on the SEM system.

A contact angle goniometer (Data Physics, OCA20, Germany) was used to measure the static water contact angle and sliding angle between a distilled water drop and the outer surface of the PVDF hollow fibre membrane. A distilled water droplet (2 µl) was deposited onto the membrane surface using a microsyringe. Each reported contact angle represents an average of five measurements taken at different positions on the membrane surface to minimize experimental errors.

Atomic force microscopy (AFM, MFP-3D-BIO) was performed in the tapping mode to examine the surface morphology of the PVDF hollow fibre membranes. The images were obtained from an area of 5.00 × 5.00 µm. Various roughness parameters obtained from the AFM analysis, such as the root mean square roughness (*R*_ms_) and maximum and minimum elevations, were used to assess the effect of the modification method.

The Fourier transform infrared spectroscopy (FTIR) spectra of the PVDF membranes were measured using attenuated total reflectance FTIR (ATR-FTIR, Nicolet 5DXC, USA).

### 2.4. Gas permeation test

The N_2_ permeation test was performed to measure mean pore size. In general, by assuming cylindrical and straight membrane pores in the membrane skin layer, the overall permeation rate of gas through a porous asymmetric membrane can be measured as the combination of Poiseuille and Knudsen flow [40]. The total permeance of gas can be calculated as follows:

2.1 $$J_{A}=\frac{2r_{p,m}\varepsilon }{3RT\;L_{P}}{\left(\frac{8RT}{\pi M}\right)}^{0.5}+\frac{r_{p,m}^{2}\varepsilon }{8\mu RT\;L_{P}}\bar{p}\;\text{or}\;J_{A}=A+B\bar{p},$$

where *J_A_* is the total gas permeance (mol m^−2^ s^−1^ Pa^−1^), *R* is the gas constant 8.314 (J mol^−1^ K^−1^), *μ* is the gas viscosity (kg m^−1^ s^−1^), *M* is the molecular weight of the gas (kg mol^−1^), *T* is the temperature of the gas (K) and $\bar{p}$ is the mean pressure (Pa).

According to equation (2.1), the mean pore size can be obtained from the intercept (*A*) and the slope (*B*) of *J*_*A*_ versus $\bar{p}$ plot as follows:

2.2 $$r_{p,m}=\frac{16B}{3A}{\left(\frac{8RT}{\pi M}\right)}^{0.5}\mu .$$

### 2.5. Measurement of overall porosity and spraying layer thickness

The overall porosity of PVDF hollow fibre membranes was determined by a commonly used method based on the gravimetric method [41]. The PVDF fibres were immersed in ethanol for 24 h and taken out to remove ethanol on the inner surface with a nitrogen stream and the wet weight was measured immediately. The porosity can be calculated as follows:

2.3 $$\varepsilon =\frac{(m_{1}-m_{2})/\rho_{1}}{(m_{1}-m_{2})/\rho_{1}+m_{2}\text{/}\rho_{2}},$$

where *m*_1_ is the weight of the dry membrane, *m*_2_ is the weight of the immersed membrane, *ρ*_1_ is the density of ethanol and *ρ*_2_ is the density of PVDF polymer.

The thickness of PVDF hollow fibre membrane can be measured from the cross-sectional SEM image. However, the thickness by spray deposition is so meagre that it cannot be calculated in this way. Assuming that the thickness and distribution of the membrane are uniform, the thickness is measured by a simple estimation. The thickness by spray deposition can be calculated as follows:

2.4 $$l=\frac{w_{1}-w_{2}}{\rho A},$$

where *l* is the thickness by spray deposition, *w*_1_ is the weight of original PVDF hollow fibre membrane, *w*_2_ is the weight of modified PVDF hollow fibre membrane, *ρ* is the SiO_2_ density and *A* is the outer surface area of PVDF hollow fibre membrane.

### 2.6. CO_2_ absorption experiment

**Figure 1.**
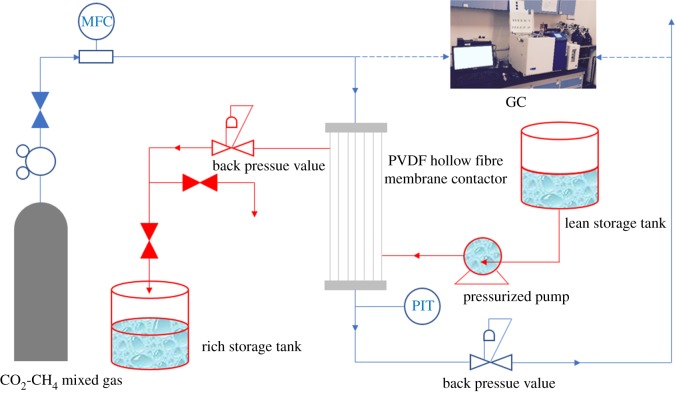
Flow diagram of experimental set-up for CO_2_ absorption.

All CO_2_ absorption experiments were carried out at room temperature using a hollow fibre membrane contactor system, as shown in figure 1. The characteristics of the unmodified and modified membrane module are listed in table 2.

**Table 2.** Specifications of the membranes used in this study solution.

parameter (unit)	unmodified membrane	modified membrane
fibre o.d. (mm)	1.1	1.2
fibre i.d. (mm)	0.8	0.8
module i.d. (mm)	15	15
effective module length	500	500
number of fibres	37	37

Pure CO_2_ or a gas mixture of 60% CH_4_ and 40% CO_2_, which is in the composition range of biogas, was fed into the tube side of the liquid–gas membrane contactor module, whereas deionized water and MEA as absorbents were circulated through the shell side.

The gas and liquid flow rates were controlled at 150 to 200 ml min^−1^ and 30 to 120 ml min^−1^, respectively, using the mass flow controllers and liquid flow meter. In the experiment, the gas operation pressure was controlled at approximately 0.11 bar lower than the liquid phase pressure to eliminate the formation of bubbles in the liquid phase, which would result in a non-uniform gas–liquid mass transfer interface [42]. The gas volumetric flow rates were measured with a soap film flowmeter. A peristaltic pump (JSX25/5 LIGAO) was used to deliver the liquid from the rich storage tank through a rotameter to the membrane contactor. A gas chromatograph (9790 Fu Li, TCD) was used to analyse the inlet and outlet concentrations of CH_4_ and CO_2_. When the CO_2_ was used as the feed gas, the CO_2_ concentration in the water outflow was measured by chemical titration. During the titration process, an excess amount of 1 M sodium hydroxide (NaOH) solution was added to the liquid sample, so that the free-dissolved CO_2_ was converted to a non-volatile ionic species. To precipitate absorbed CO_2_ as BaCO_3_, an excess amount of 10 wt% BaCl_2_ solution was added to the aforementioned solution. The excess NaOH was titrated with 1 M HCl solution using phenolphthalein as the indicator. When the titration of previous stage was over, in order to measure the amount of BaCO_3_, the HCl solution was then added to using methyl orange as the second indicator. All experimental results were collected after the system reached the steady state (less than 10 min).

In this study, CO_2_ absorption flux was used to evaluate the separation properties of the hollow fibre membrane module, which can be calculated by the following equation [43]:

2.5 $$J_{{\mathrm{CO}}_{2}}=\frac{(Q_{g,\mathrm{in}}\;\times \;C_{g,\mathrm{in}}-Q_{g,\mathrm{out}}\;\times \;C_{g,\mathrm{out}})\;\times 273.15\;\times 1000}{22.4\;\times \;T\;\times \;S},$$

where $J_{{\mathrm{CO}}_{2}}$ is the CO_2_ absorption flux [mol (m^2^ s)^−1^]. *C*_g,in_ and *C*_g,out_ are the inlet and outlet gas–phase CO_2_ concentrations, respectively (%). *Q*_g,in_ and *Q*_g,out_ are the gas volumetric flow rate at the inlet and outlet, respectively (m^3^ s^−1^). *T* is the temperature (K); and *S* is the gas–liquid interfacial area (m^2^).

### 2.7. Membrane mass transfer measurement

**Figure 2.**
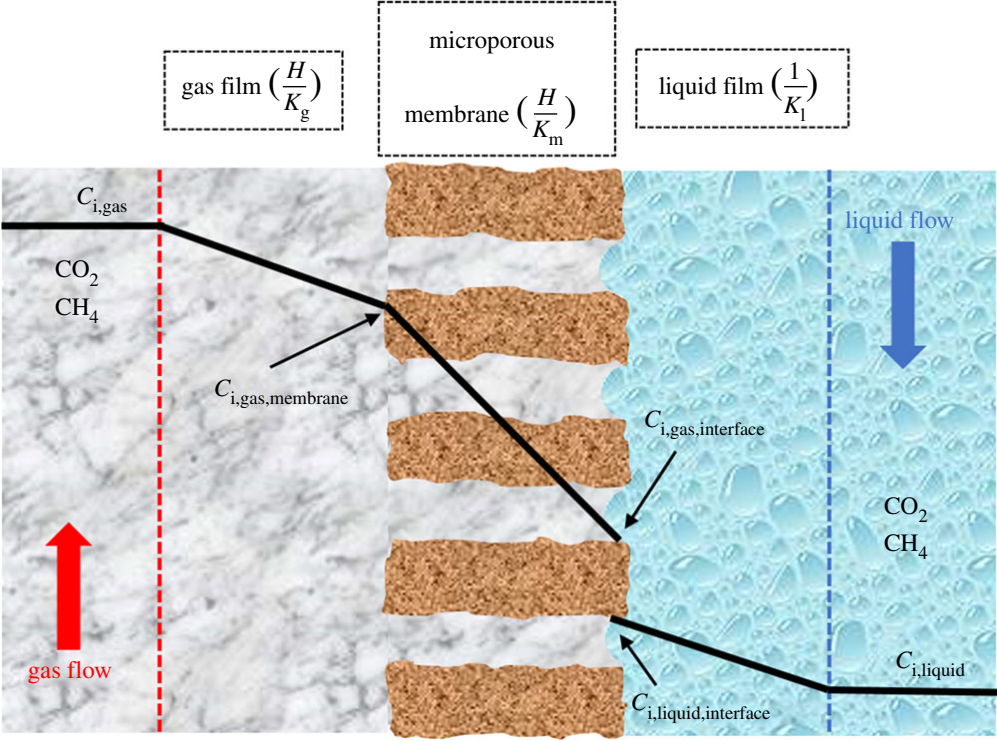
A schematic drawing of the resistance-in-series based on film theory in non-wetted membrane contactor.

In order to estimate the membrane mass transfer resistance, the Wilson plot method was used [44]. Figure 2 shows the mass transport of the hydrophobic hollow fibre membrane with non-wetted pores. As can be seen, the gas of interest has to diffuse from the gas phase boundary layer (*k*_g_, m s^−1^) through the membrane (*k*_m_, m s^−1^) and into the liquid phase boundary layer (*k*_l_, m s^−1^); the overall mass transfer resistance (*K*_O_^−1^) can be expressed by the resistance-in-series model, written as equation (2.2) [44].

2.6 $$\frac{1}{K_{O}}=\frac{1}{k_{l}}+\frac{Hd_{\mathrm{out}}}{k_{m}d_{\ln}}+\frac{Hd_{\mathrm{out}}}{k_{g}d_{\mathrm{in}}},$$

where *d*_in_, *d*_out_ and *d*_ln_ are the inner diameter (m), outer diameter (m) and logarithmic mean diameter (m) of fibres, respectively. *H* is Henry's constant and is 0.85 for water [45]. By considering pure CO_2_ as the feed gas, the gas phase mass transfer resistance can be neglected. Hence, the total mass transfer process is determined only by the membrane and liquid mass transfer resistances. There is a proportional relationship between liquid mass transfer resistance and liquid velocity ${V_{l}}^{-\alpha }$, where α is an empirical constant. The drawing of 1/*K*_o_ versus ${V_{l}}^{-\alpha }$ results in a straight line, which is called a Wilson plot. The value of α is chosen based on the fact that it provides the best straight line through the data points. The membrane's mass transfer resistance can be calculated using the intercept of the Wilson plot. The overall mass transfer coefficient, *K*_0_, is expressed as follows [46]:

2.7 $$K_{0}=-\frac{Q_{l}}{S}\text{ln}\left(1-\frac{C_{l,\mathrm{out}}}{H\;C_{g}}\right),$$

where *Q*_l_ is the water volumetric flow rate (m^3^ s^−1^), *S* is the gas–liquid interfacial area (m^2^) and *H* is Henry's constant, which is 0.85 for water [45]. *C*_g_ and *C*_l,out_ are the gas-phase and liquid-phase outlet CO_2_ concentrations of the module, respectively (mol m^−3^).

## 3. Results and discussion

### 3.1. Morphological structure of coatings

**Figure 3.**
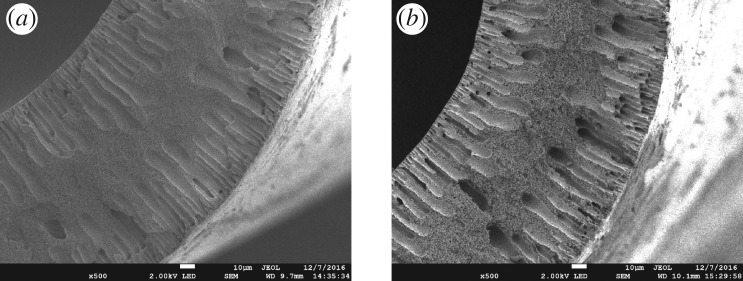
Cross-sectional SEM images of (*a*) B0 and (*b*) B2.

**Figure 4.**
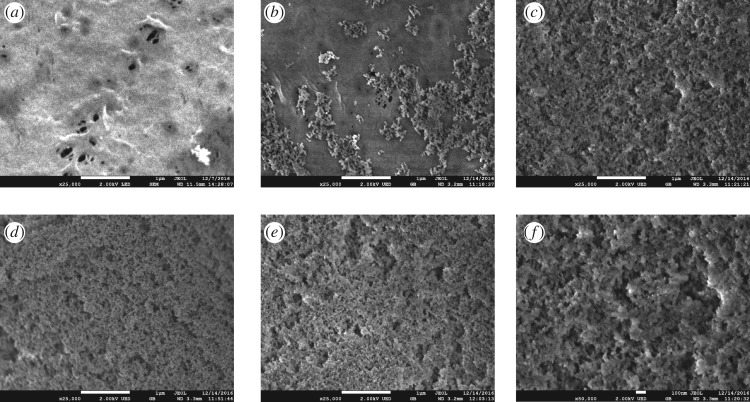
Top-surface SEM images depicting the change in morphology of (*a*) B0, (*b*) B1, (*c*) B2, (*d*) B3, and (*e*) B4 (25 000×); (*f*) magnified image of (*c*) (50 000×).

As mentioned previously, surface hydrophobicity is determined by the low-energy molecules of a surface and its well-defined roughness. SEM micrographs of the cross sections of the unmodified and modified membranes are presented in figure 3. As can be seen in figure 3*a* and *b*, both the fibre membranes consist of an intermediate layer with a sponge-like structure and a sublayer with finger-like microporous structure originating from the middle part of the membrane and extending to the top and bottom layers of the fibre membrane. The SEM images in figure 4 show the surface morphologies of the unmodified and modified hollow fibre membranes. The unmodified membrane is denoted as B0, and the modified membranes prepared by spraying suspensions containing 0.08, 0.2, 0.4 and 0.6 (g/45 ml) of silica are referred to as B1, B2, B3 and B4, respectively. Low-magnification SEM images show that the surface coating is formed by the aggregation of silica nanoparticles. Comparing figure 4(*b*), (*c*), (*d*) and (*e*), it appears that the morphologies of the modified membranes are affected by the SiO_2_ nanoparticle content. The micro-level silica roughness became dense as the silica nanoparticle content increased, thus forming a nearly continuous roughness (figure 4*c*). The presence of nanoscale roughness is evident from the high-magnification SEM image (figure 4*f*) and is due to the presence of nanosized silica particles in the coating. It can be seen from the SEM image (figure 4*f*) that the size of the R106 aggregates on the membrane surface is about 30 nm. As established previously, a combination of multi-level structure and low-energy materials ensures superhydrophobicity [47,48]. When the gas molecules fill the gap in the silica layer of the membrane, air pockets would form, and thus, the solid–liquid contact area would reduce [49]. Therefore, the droplet cannot spread on the micropores, and the hierarchical roughness is beneficial to the surface superhydrophobicity [50]. It can be deduced from figures 4*f* and 6*b* that thicker but more homogeneous coatings can be achieved by increasing the amount of silica in the sprayed suspension, compared with figure 4*b* and *d*. Although a uniform distribution of SiO_2_ nanoparticles is good for surface superhydrophobicity, the silica coating increased the surface mass transfer resistance. Consequently, it was necessary to carry out the liquid–gas membrane separation experiment based on this experiment to determine the best spraying conditions.

**Figure 5.**
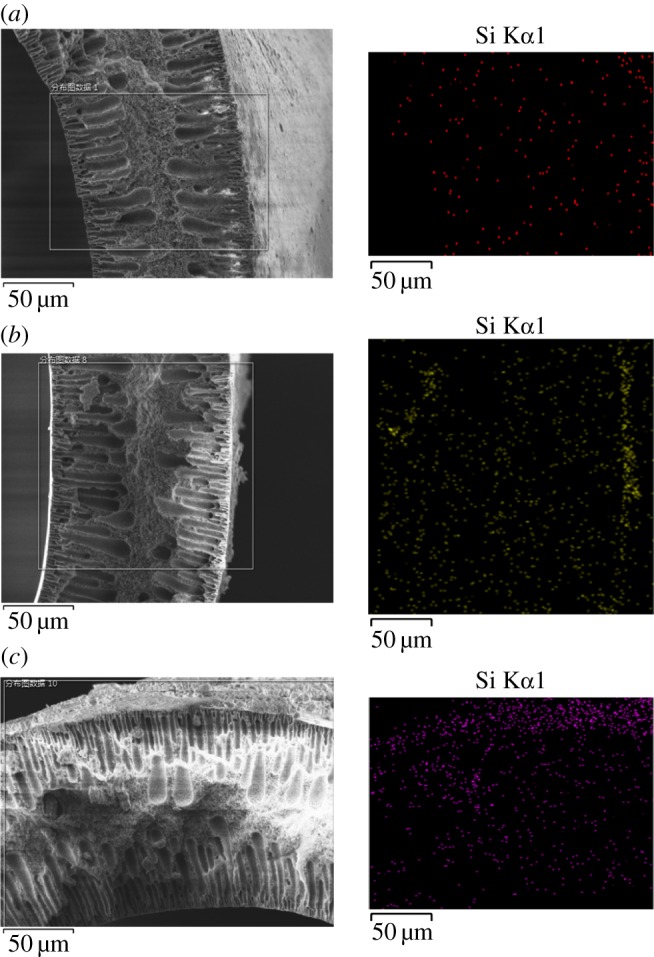
EDS elemental analysis of silicon distribution in (*a*) B0, (*b*) B2, and (*c*) B4 hollow fibre membrane cross sections.

Figure 5 shows the results of EDS analyses of the distribution of silica nanoparticles inside the membrane. The coloured spots on the dark background represent the position of silica on the cross sections of the PVDF membranes. As can be seen, a little silica penetrated into the membrane pores during the spraying process. It can also be observed that the distribution of silica on the outer surface became denser as the silica content in the spray solution increased.

### 3.2. Membrane surface analysis

**Figure 6.**
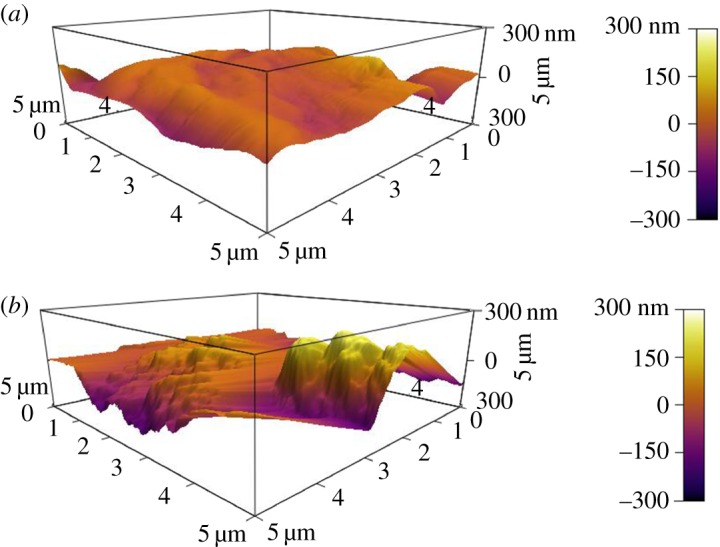
AFM images of 5 × 5 µm surfaces of (*a*) pristine PVDF (B0) and (*b*) coated PVDF (B2).

AFM was used to measure the nano-level roughness of the PVDF hollow fibre membrane, as indicated by SEM. Owing to the tip dilation effect, the morphology of AFM can be larger than that measured by SEM. The morphology as measured by AFM is only a reference value; the specific morphology should be based on SEM measurement. As presented in table 3, the *R*_ms_ value of the original membrane is 33 nm and that of the modified membrane is 64 nm. This is because a large number of silica aggregates formed on the surface of the PVDF hollow fibre membranes (figure 6*b*). The aggregates on the membrane surface appear as bright protrusions, whereas the holes appear as dark areas in the image. It can be deduced that the silica coating resulted in homogeneous roughening, thus forming a superhydrophobic membrane. In addition, maximum elevation is maximum peak height within the analysed area with respect to the mean data plane, minimum elevation is lowest data point in examined region, and the mean data plane is the reference point defined here.

**Table 3.** Roughness parameters of pristine PVDF and coated PVDF as measured by AFM.

membrane	amount of SiO_2_ nanoparticles (g) [concentration (mg PVDF/ml solution)]	*R*_ms_ (nm)	maximum elevation (nm)	minimum elevation (nm)
B0	0 [0]	33	115	−165
B1	0.08 [1.78]	50	202	−172
B2	0.2 [4.44]	64	253	−195
B3	0.4 [8.89]	65	254	−193
B4	0.6 [13.33]	65	252	−196

The hydrophobicity of the surface layer can be determined by measuring the water contact angle of the hollow fibre membrane. Table 4 shows the variation in contact angle and sliding angle with SiO_2_ nanoparticle content. As shown in table 4, when the SiO_2_ nanoparticle content increased from 0 g/45 ml to 0.2 g/45 ml, the water contact angle dramatically increased from 92° to 150° and sliding angle decreased from 80° to 3°. However, with a further increase in silica concentration, the water contact angle and sliding angle did not change much. This change can be easily interpreted in accordance with the surface morphology. As shown in figure 4, when the SiO_2_ nanoparticle content is <0.2 g/45 ml, the distribution of silica aggregates on the membrane surface is not uniform. When the SiO_2_ nanoparticle content reaches 0.2 g/45 ml, the SiO_2_ nanoparticles form a uniformly deposited layer on the membrane surface (figure 4*c*). The structure of the surface greatly improves the drainage of water, so the water drops falling on the membrane surface tend to roll off. This result indicates that the modified PVDF hollow fibre membrane has superhydrophobic properties. The superhydrophobic phenomena can be explained with the Wenzel and Cassie models [18,51]. When a droplet deposited on a solid surface is surrounded by gas, a contact angle (*θ*) is formed at the gas–liquid interface. According to the Wenzel model, *θ* changes to an apparent contact angle (*θ*^e^) when the liquid flows to the lower voids, thus increasing the liquid–solid contact area [18]. In the Wenzel model, roughening transforms a hydrophilic surface into a superhydrophilic surface; however, if the surface is hydrophobic with a water contact angle greater than 90°, roughing renders it superhydrophobic. The relationship between *θ*^e^ and *r* in Wenzel's model is shown in the following equation:

3.1 cos ⁡θe=rcos ⁡θ.

If the surface is complex and the liquid has sufficient capillary force, the liquid will not penetrate into the texture of the rough surface, in which case the Wenzel hypothesis will be violated. However, this concept can be introduced with the Cassie model wherein the air–liquid–solid interface considers the air gap below the droplet, and is expressed as follows:

3.2 cos ⁡θe=fs(cos ⁡θ+1)−1.

**Table 4.** Characteristics of original and modified PVDF hollow fibre membranes.

membrane type	contact angle (°)	sliding angle (°)	spraying layer thickness (µm)	overall porosity (%)	mean pore size (µm)
B0	92 ± 1	80 ± 4	0	70.11 ± 0.85	0.2
B1	145 ± 3	7 ± 1	0.87 ± 0.4	70.51 ± 0.93	0.198
B2	155 ± 3	<3 ± 1	1.77 ± 0.4	71.01 ± 0.89	0.196
B3	155 ± 3	<3 ± 1	1.87 ± 0.4	71.12 ± 0.97	0.194
B4	155 ± 3	<3 ± 1	1.93 ± 0.4	71.18 ± 0.95	0.193

From the above equation, it is evident that if the solid–liquid contact area fraction *f*_s_ decreases, *θ*^e^ will increase, and when the fraction *f*_s_ approaches 0, *θ*^e^ approaches 180° [51]. In other words, an increase in roughness results in a decrease in solid–liquid contact area, so that the contact angle increases. As shown in tables 3 and 4, an increase in *R*_ms_ roughness led to an increase in the water contact angle.

**Figure 7.**
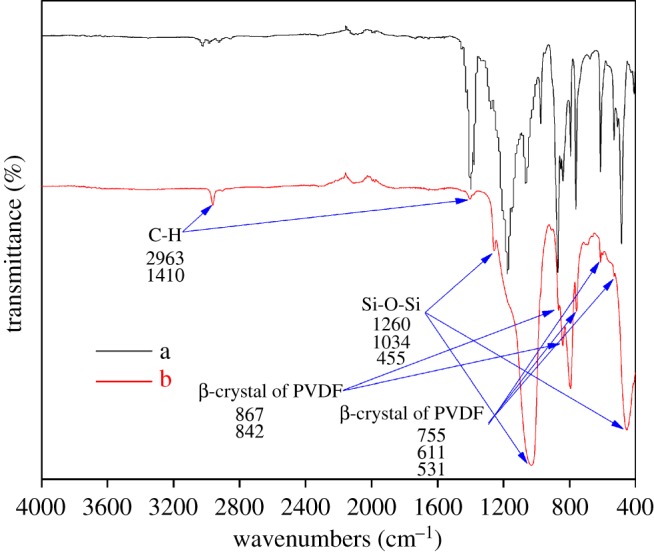
FTIR spectra of (a) B0 and (b) B2.

Figure 7 shows the FTIR spectra of unmodified and modified (with 0.2 g/45 ml R106) PVDF hollow fibre membranes. The pristine PVDF hollow fibre membrane exhibits typical bands around 867 and 755 cm^−1^ and characteristic peaks at 755, 611 and 531 cm^−1^, which correspond to the β-crystal and α-crystal structures of PVDF, respectively. Compared to the unmodified fibre membrane, three new absorption bands appeared at 1260, 1034 and 455 cm^−1^, which are attributed to the bending vibrations and symmetric stretching modes of Si-O-Si [39,52,53]. Furthermore, additional FTIR peaks and their assignations are listed in table 5.

**Table 5.** Assignations of FTIR peaks of (a) B0 and (b) B2.

wavenumber (cm^−1^)	functional groups
2963	C-H stretching
1410	C-H deformation
1260, 1034	asymmetric Si-O-Si stretching
867, 842	β-crystal of PVDF
755, 611, 531	α-crystal of PVDF
455	Si-O-Si bending

**Figure 8.**
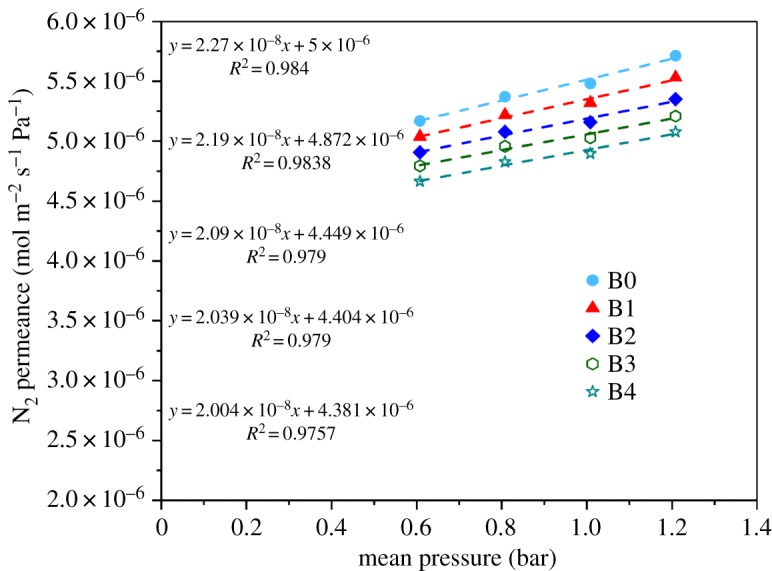
N_2_ permeation test results for PVDF membranes.

Figure 8 reveals the permeance rate of N_2_ (mol m^−2^ s^−1^ Pa^−1^) versus mean pressure (bar). In this figure, linear fits to the data resulted in broken lines with reasonably high correlation coefficients, *R*^2^. Also, the value of the average pore size was calculated using these data according to equation (2.2). The results obtained are provided in table 4. As shown in table 4, the average pore sizes of the original and modified membranes are almost the same. Also as noted in table 4, the value of porosity for the modified membrane was slightly different from the unmodified one, indicating that the modified membrane still has good porosity. In addition, the slight difference in porosities among the four kinds of modified membranes may be due to the nature of the silica nanoparticle deposition layer. The thickness of the sprayed layer is also shown in table 4, and it can be seen that the thickness of the sprayed layer increases with increasing SiO_2_ nanoparticle content.

### 3.3. CO_2_ absorption results

**Figure 9.**
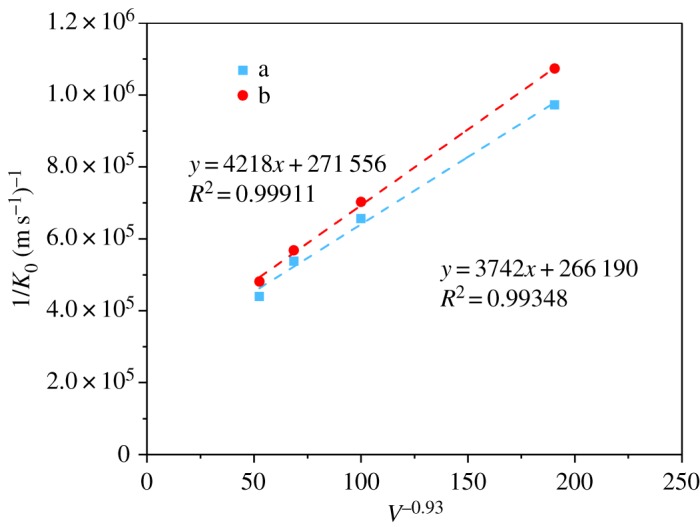
Wilson plot of the hollow fibre membrane (pure CO_2_-distilled water system): (a) B0 and (b) B2.

The effect of different membranes on the system performance can be shown quantitatively by the membrane resistance. The Wilson plot of 1/*K*_0_ versus *V*^−0.93^ was obtained, as shown in figure 9. The *V*^−0.93^ was confirmed to represent the best linear fit to the data of the Wilson plot. Yang and Cussler also used the relation of 1/*K*_0_ with *V*^−0.93^ to describe gas–liquid membrane absorption, where the liquid was in the shell side of the membrane module [54]. The membranes' mass transfer resistance was calculated using the intercept with the *y*-axis of the Wilson plot in figure 8.

As the liquid-phase resistance is dominant in the case of physical absorption, the overall mass transfer resistance considerably decreases by increasing the absorbent velocity. It can be connected with the smaller boundary layer thickness at a higher velocity of the liquid, which can minimize the liquid-phase resistance [11]. With the spray deposition of SiO_2_ on the PVDF hollow fibre membrane surface, the membrane mass transfer resistance increased slightly: 266 190 and 271 556 s m^−1^ for the original and modified membranes, respectively.

At the liquid flow rate of 120 ml min^−1^ (≈0.014 m s^−1^), the membrane mass transfer resistance contributed 24.8% of the total resistance for the original membrane, whereas its contribution increased to 25.3% for the modified membrane.

### 3.4. Long-term CO_2_ absorption performance of original and modified PVDF membrane

**Figure 10.**
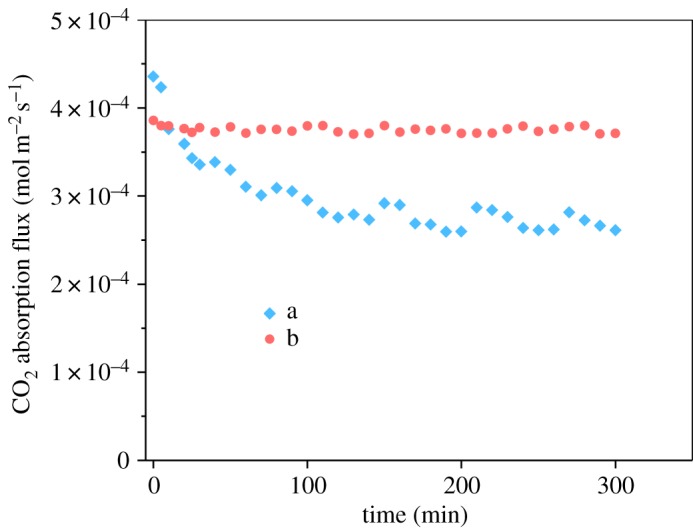
Influence of modification of the PVDF membrane on CO_2_ mass transfer rate during long-term operation (*Q*_in,gas_: 200 ml min^−1^; *Q*_in,liquid_: 60 ml min^−1^; *C*_in,CO_2__: 40%; absorbent: 0.1 mol l^−1^ MEA, at 0.11 MPa liquid net pressure) of (a) B0 and (b) B2.

In order to study the effect of membrane modification on membrane flux, continuous experiments over 5 h were conducted. The inlet gas contained CO_2_ 40%, and CH_4_ was used as the equilibrium gas. The gas flow rate and the liquid flow rate were 200 ml min^−1^ and 30 ml min^−1^, respectively. In the membrane gas contactor operation, CO_2_ was fed through the tube side and the liquid absorbent flowed on the shell side of the membrane module. Figure 10 shows the experimental results using 0.1 M MEA as an aqueous solution (at 0.11 MPa liquid net pressure). The CO_2_ absorption flux decreased by approximately 28% after 80 min of operation when the CO_2_ absorption experiment was carried out using the unmodified membrane contactor, and the carbon dioxide CO_2_ absorption flux did not change significantly until the end of operation. The reduction in the CO_2_ absorption flux came from the partial wetting of the membrane. As PVDF is a hydrophobic material and the pore size of the PVDF membrane is small, the MEA solution is unlikely to penetrate into the membrane pores. It is possible that the capillary condensation of the MEA solution in the membrane pores leads to partial wetting of PVDF membrane, because the Kelvin equation predicts that unsaturated water vapour is more prone to capillary condensation in channels of adequately small size [55]. A similar deterioration of flux was reported, by Sadoogh *et al*. [57], for the PVDF membranes in CO_2_ absorption in a diethanolamine (DEA) solution [56]. But, the capillary condensation of the MEA solution in the membrane pores will happen to modify PVDF hollow membranes too. Based on the SEM image, it appears that the surface of PVDF hollow fibres has several large pores, which were covered by Silica/PDMS coating after modification. And 5 h CO_2_ absorption results clearly showed that the flux did not further drop after 3 h; therefore, some water/MEA might get into those large pores shown in the original PVDF hollow fibre SEM image (figure 4*a*) and block those pores gradually in the first 3 h (at 0.11 MPa liquid net pressure), but after 3 h, the rest of the pores have sufficiently small diameter and enough liquid entry pressure to prevent further wetting.

Compared with wetted mode (liquid-filled pores), non-wetted mode is more conducive to the absorption of acid gas in GLMC, because the gas diffusion coefficient is higher in non-wetted mode. When the penetration pressure is larger than the pressure difference between the gas and liquid stream in the membrane pores, the porous membrane will not permit absorbents to enter the pores. This phenomenon can be estimated using the Laplace–Young equation:

3.3 ΔP=−2σcos ⁡θrp,

where Δ*P* is the penetration pressure or wetting pressure (KPa), *σ*_L_ is the surface tension of the liquid (mN m^−1^), *θ* is the contact angle between the liquid phase and the membrane (°) and *r*_P_ is the membrane pore size (m).

Based on the SEM image, it appears that the surface of PVDF hollow fibres has several larges pores, which were covered by silica/PDMS coating after modification. Therefore, the value of *r*_p_ for original PVDF fibres is higher than that of modified PVDF fibres. In addition, the value of contact angle for original PVDF fibres is smaller than that of modified PVDF fibres (table 4). Combining with equation (2.5), it can be concluded that the penetration pressure for original PVDF fibre is lower than that for modified PVDF fibre, and the lower in the liquid penetration pressure is caused by those large defects shown in SEM images (figure 4*a*). Therefore, the gradual decrease of CO_2_ absorption flux in original PVDF hollow fibres can be caused by the larges pores appearing in the surface of PVDF hollow fibres.

In the case of the modified PVDF membrane contactor, the decrease of CO_2_ flux was not appreciable at approximately 96% of the initial value. As mentioned previously, the unmodified membrane was easily wetted during long-term operation of gas–liquid membrane separation. As modified PVDF membranes are more hydrophobic than original membranes, the modified membranes have higher stability and durability. It was noted that in the initial 10 min, the modified membrane had a slightly lower CO_2_ flux than the modified membrane. Although the modified membranes have superhydrophobic properties, this advantage cannot overcome the increase in membrane thickness, which is detrimental to the increase in CO_2_ flux. After 10 min, the original membranes were further wetted, and the modified membrane contactor exhibited a higher CO_2_ absorption flux than that of the unmodified membrane contactor. The superhydrophobic properties reduced the sensitivity of the modified membrane to wetting, which reduced the negative aspect of increased mass transfer resistance caused by modification. The analysis described in the present study demonstrates that a PVDF hollow fibre membrane with a superhydrophobic layer has the potential to improve the stability of long-term performance in liquid–gas membrane processes.

**Figure 11.**
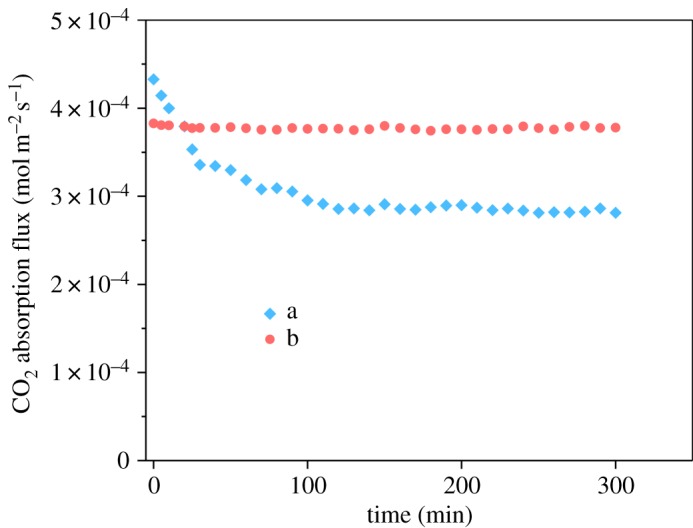
Influence of modification of the PVDF membrane on CO_2_ mass transfer rate during long-term operation (*Q*_in,gas_: 200 ml min^−1^; *Q*_in,liquid_: 60 ml min^−1^; *C*_in_,_CO_2__: 40%; absorbent: 0.1 mol l^−1^ MEA, at 0.05 MPa liquid net pressure) of (a) B0 and (b) B2.

Figure 11 shows the long-term CO_2_ absorption performance of original and modified PVDF membrane using 0.1 M MEA as an aqueous solution (at 0.05 MPa liquid net pressure). Liquid flow rate was fixed at 30 ml min^−1^. Gas flow rate was fixed at 200 ml min^−1^. The inlet gas contained CO_2_ 40% in the balance of CH_4_. By comparing figures 10 and 11, we can find that the difference in flux before and after modification can be smaller with lower pressure difference. That is because less water/MEA gets into those large defects shown in the original PVDF hollow fibre SEM image.

In some cases, the absorbents temperature is higher than room temperature, so it is necessary to carry out the gas separation experiment at high temperature. However, due to laboratory conditions and financial constraints, we cannot install the temperature control device on the experimental equipment, so we could not complement the study on membrane behaviour during elevated temperature in this paper. Results obtained by Ismail *et al*. found that a significant increase in the CO_2_ flux was observed by decreasing the absorbent temperature [58]. However, in the case of chemical absorption, the reaction rate was dominant, where the CO_2_ flux was significantly increased by increasing the absorbent temperature. Furthermore, Yan *et al.*'s study showed that a significant increase of CO_2_ flux with temperature (from 30 to 50°C) for chemical absorption of CO_2_ using methyldiethanolamine (MDEA) in the PP hollow fibre membrane contactor [43].

**Figure 12.**
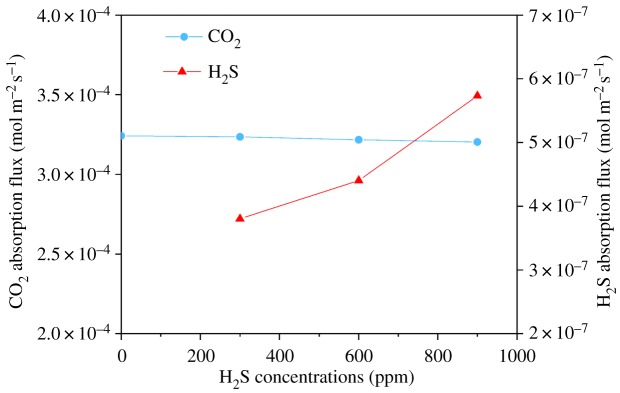
Effect of H_2_S concentration on CO_2_ and H_2_S absorption flux (gas flow rate was fixed at 200 ml min^−1^, liquid flow rate: 30 ml min^−1^, feed gas contained CO_2_ 40%v/v and H_2_S 0 to 900 ppm in the balance of CH_4_).

Figure 12 shows the influence of H_2_S concentration on CO_2_ and H_2_S flux when using 0.1 M MEA as the absorbent solution. Liquid flow rate was fixed at 30 ml min^−1^. Gas flow rate was fixed at 200 ml min^−1^. The inlet gas contained CO_2_ 40%, and the H_2_S concentrations varied between 0 and 900 ppm (for biogas) in the balance of CH_4_. The H_2_S absorption flux in this system increases with the increase of H_2_S concentration. In addition, the change of H_2_S concentration in the gas phase did not significantly influence CO_2_ absorption flux. In the current work, the concentration of H_2_S is much lower than CO_2_ (CO_2_/H_2_S about 450 times). Hence, the mass transfer process of CO_2_ was not influenced by the change of H_2_S concentration. Similar experimental results were reported in our previous work in Jin *et al*. [59].

**Figure 13.**
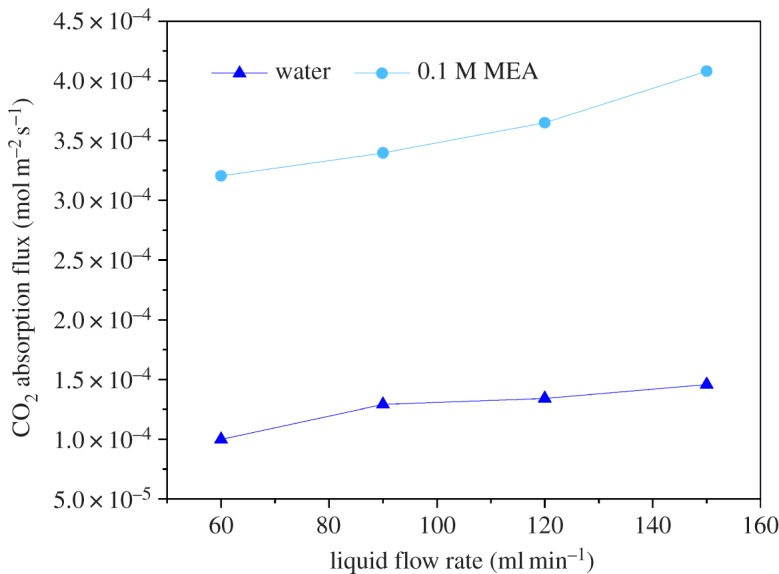
Effect of liquid flow rate and types of solutions on CO_2_ absorption flux (gas flow rate: 200 ml min^−1^, feed gas contained CO_2_ 40%v/v and H_2_S 300 ppm in the balance of CH_4_).

Figure 13 represents the effect of liquid flow rate and types of solutions on CO_2_ and H_2_S flux for water and 0.1 M MEA. The inlet gas contained CO_2_ 40%v/v in the balance of CH_4_. Gas flow rate was fixed at 200 ml min^−1^. Liquid flow rate was varied from 30 to 120 ml min^−1^. In the range of liquid absorbent flow rate studied (30–120 ml min^−1^), it is clear that the separation efficiency of water is lower than that of chemical absorption (MEA as chemical absorbent). This is because the effect of physical absorption is determined by the solubility of acid gas in physical absorbents. While using MEA solution, CO_2_ can react chemically with MEA in the liquid phase, and the removal efficiencies were increased. Figure 13 also shows that the increase of the liquid flow rate had a good effect on the increase of CO_2_ absorption flux. A number of previous studies have reported a similar conclusion, namely that the mass transfer process of CO_2_ absorption is controlled by the liquid phase in GLMC [45,60].

## 4. Conclusion

Superhydrophobic PVDF hollow fibre membranes with a water contact angle of 155° ± 3° were prepared by coating the membranes with a suspension of commercially available hydrophobic SiO_2_ nanoparticles at room temperature and atmospheric pressure. The hollow fibre membrane's hydrophobicity and homogeneity could be controlled by varying the silica nanoparticle content. A PVDF hollow fibre membrane with a remarkably high water contact angle was obtained by optimizing the content of silica particles in the sprayed suspension. The SEM and AFM results revealed that the superhydrophobicity of the PVDF hollow fibre membrane resulted from micro/nano binary roughness. In the continuous CO_2_ absorption test, the modified superhydrophobic PVDF membrane showed more stable and efficient performance than the original PVDF membrane. In conclusion, a superhydrophobic PVDF hollow fibre membrane modified by the method of spray deposition is a promising alternative for gas–liquid membrane contactor systems in terms of long-term operation.

## Data accessibility

All theoretical calculations presented herein were performed with the equations developed in this paper. Our data have been deposited in the Dryad Digital Repository: (http://dx.doi.org/10.5061/dryad.5qt15) [61].
